# Prediction of the Pharmacokinetics, Pharmacodynamics, and Efficacy of a Monoclonal Antibody, Using a Physiologically Based Pharmacokinetic FcRn Model

**DOI:** 10.3389/fimmu.2014.00670

**Published:** 2015-01-05

**Authors:** Manoranjenni Chetty, Linzhong Li, Rachel Rose, Krishna Machavaram, Masoud Jamei, Amin Rostami-Hodjegan, Iain Gardner

**Affiliations:** ^1^Simcyp Limited (a Certara Company), Sheffield, UK; ^2^Manchester Pharmacy School, Manchester University, Manchester, UK

**Keywords:** monoclonal antibodies, FcRn model, PBPK models, PBPK/PD, efalizumab

## Abstract

Although advantages of physiologically based pharmacokinetic models (PBPK) are now well established, PBPK models that are linked to pharmacodynamic (PD) models to predict pharmacokinetics (PK), PD, and efficacy of monoclonal antibodies (mAbs) in humans are uncommon. The aim of this study was to develop a PD model that could be linked to a physiologically based mechanistic FcRn model to predict PK, PD, and efficacy of efalizumab. The mechanistic FcRn model for mAbs with target-mediated drug disposition within the Simcyp population-based simulator was used to simulate the pharmacokinetic profiles for three different single doses and two multiple doses of efalizumab administered to virtual Caucasian healthy volunteers. The elimination of efalizumab was modeled with both a target-mediated component (specific) and catabolism in the endosome (non-specific). This model accounted for the binding between neonatal Fc receptor (FcRn) and efalizumab (protective against elimination) and for changes in CD11a target concentration. An integrated response model was then developed to predict the changes in mean Psoriasis Area and Severity Index (PASI) scores that were measured in a clinical study as an efficacy marker for efalizumab treatment. PASI scores were approximated as continuous and following a first-order asymptotic progression model. The reported steady state asymptote (*Y*_ss_) and baseline score [*Y* (0)] was applied and parameter estimation was used to determine the half-life of progression (*T*_p_) of psoriasis. Results suggested that simulations using this model were able to recover the changes in PASI scores (indicating efficacy) observed during clinical studies. Simulations of both single dose and multiple doses of efalizumab concentration-time profiles as well as suppression of CD11a concentrations recovered clinical data reasonably well. It can be concluded that the developed PBPK FcRn model linked to a PD model adequately predicted PK, PD, and efficacy of efalizumab.

## Introduction

Binding to the neonatal Fc receptor (FcRn) as well as therapeutic targets *in vivo* have a significant influence on the disposition of a monoclonal antibody (mAb). Physiologically based pharmacokinetic (PBPK) models describing some of these processes in the disposition of mAbs in pre-clinical species and humans, have recently been published (1–6). PBPK models are mechanistic and are regarded as a more realistic representation of drug disposition *in vivo*. However, the ultimate interest in mAbs is their therapeutic potential and the majority of current PBPK models have not advanced to include prediction of response to mAbs. A PBPK model linked to a pharmacodynamics model (PD) will offer the advantage of predicting both the PK variability and the response to a mAb. In addition, input to the PD model can be done from a tissue interstitial compartment and not just from plasma, which is important when modeling membrane bound receptors.

The PKs of mAbs are complex and have been reviewed (7–11). Key aspects of PBPK models for mAbs represent the tissue physiology and quantitation of the distribution, metabolism, and elimination of mAbs. These include characterization of the tissue structure to account for the vascular space, interstitial space (where the drug target can be located), and the endosomal space (where non-specific catabolism of the mAb may occur); distribution of mAbs by diffusion and convection (extravasation); binding of immunoglobulin G (IgG) to FcRn to account for protection of mAbs from degradation, as well as specific high-affinity catabolism (receptor-mediated endocytosis) due to binding of the mAb to a specific target, i.e., target-mediated drug disposition (TMDD), where relevant. Binding of the mAb to the specific target may also produce the PD effects and pharmacological response to the mAb, which varies with dynamic changes in the availability of the free target sites. The latter feature of mAbs can be characterized using PBPK models linked to PD models.

Psoriasis is a chronic inflammatory skin disorder with a proliferation of keratinocytes and accumulation of activated T cells in skin lesions. This incurable autoimmune disease is characterized by lesions in the epidermis and dermis and is mediated by T lymphocytes. Leukocyte-function associated-antigen type 1 (LFA-1) is a T lymphocyte adhesion molecule, which plays a crucial role in the pathogenesis of psoriasis. Efalizumab is a recombinant humanized IgG1 mAb that inhibits T-cell adhesive interactions by binding to CD11a, which is the alpha subunit of LFA-1. Blockade of the CD11a target results in the interruption of the immunological cascade responsible for psoriatic plaque formation (12, 13).

Response of psoriasis to a therapeutic intervention can be assessed using the Psoriasis Area and Severity Index (PASI) score. Several clinical studies that have evaluated the PK and response to different doses of efalizumab in psoriasis have been published (12, 14–18), providing adequate clinical data for the development and verification of a PBPK model linked to a PD model. Thus, although efalizumab is no longer used therapeutically (due to toxicity), it is used to illustrate the application of the PBPK/PD model in this study.

The aim of this study was to develop a PD model that is linked to the Mechanistic FcRn Model for mAbs within the Simcyp Population Based Simulator to simulate the efficacy of efalizumab in patients with plaque psoriasis.

## Materials and Methods

### PBPK model to characterize mAb PK and TMDD

**Figure 1:**
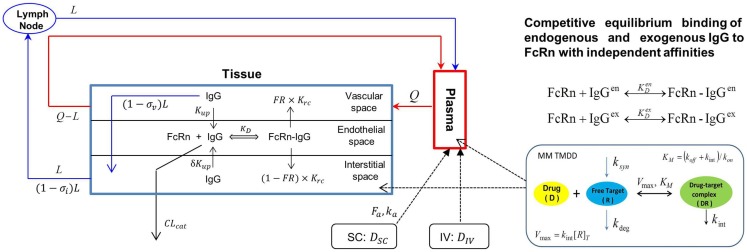
**Model structure of the physiologically based Mechanistic FcRN model for mAbs coupled with a Michaelis–Menten based target-mediated drug disposition (MM TMDD) model**. Q – plasma flow rate, L – lymph flow rate, *K*_up_ – endothelia uptake rate, σ_v_ – vascular reflection coefficient, σ_i_ – lymphatic reflection coefficient, *K*_rc_ – rate of recycling of bound IgG, FR – recycling fraction of FcRn-bound IgG, δ – ratio of uptake rates in luminal and abluminal sides, *K*_D_ – equilibrium dissociation constant for IgG and FcRn binding, CL_cat_ – intrinsic clearance of IgG, [*R*]_T_ – total target concentration, *k*_syn_ – first-order synthesis rate constant of target, *k*_deg_ – zero-order degradation rate of target, *k*_int_ – internalization rate constant of complex, *k*_on_ – association rate constant of drug and target, *k*_off_ – dissociation rate constant of drug–target complex. Superscripts “en” and “ex” represent endogenous and exogenous IgGs, respectively.

The Simcyp population based simulator (Version 13 R1) was used to construct a PBPK model with TMDD for efalizumab, in a virtual north European Caucasian population. Parameters used in the efalizumab compound file are listed in Table 1 (19–21). The Mechanistic FcRn model, which accounts for disposition of mAbs in the vascular, endothelial, and interstitial layers within the tissue compartment, was used (22) (Figure 1). Key features of this model include:

- Transport of mAbs through pores in the endothelial cells and lymph vessels by convection (modeled using reflection coefficients).
- Competitive binding of exogenous (efalizumab) and endogenous IgG to FcRn in the endothelial space. FcRn-bound IgG is transported to either the vascular (recycling) or interstitial layers (transcytosis).
- Catabolism of free IgG (unbound to FcRn) in the lysosomes of the endothelial space.

**Table 1:** **Parameter values used for efalizumab in the Simcyp Simulator**.

Parameter	Value	Reference/comments
MW: molecular weight of efalizumab	148841 g/mol	(19)
*K*_D_: equilibrium binding constant from FcRN	2.96423 μM	Estimated using linear regression and the relationship between half-life and *K*_D_ as per Suzuki et al. (21)
CLiv: clearance	0.0227 l/h; CV% = 30	(20)
Molecular weight of target CD11a	150000 Da	(20)
*K*_deg_: degradation rate of the target ieCD11a	0.0185 1/h; CV% = 10	(4)
*K*_m_: rate constant for receptor complex internalization and degradation	0.000573 μM	(20)
*K*_int_: internalization rate constant for the complex	0.1 l/h	(18)
Rmax: CD11a abundance	0.01 μM	Estimated
*K*_syn_: rate of synthesis of target	0.000185 μM/h	*K*_syn_ = *R*_max_*K_deg_

The PD effects of efalizumab on CD11a free concentrations as well as the catabolism of efalizumab resulting from binding to CD11a was quantified using a Michaelis–Menten (MM) approximation of the TMDD model as described by Gibiansky and coworkers (23) (Figure 1), which accounted for the following:

- Binding of efalizumab to free CD11a characterized by *k*_on_.
- Dissociation of the efalizumab–CD11a complex (*k*_off_).
- Internalization or degradation of the complex (*k*_int_).
- Saturation of CD11a binding sites.
- Changes in CD11a concentration due to synthesis (*K*_syn_) and degradation (*K*_deg_).
- *K*_m_ (rate constant for receptor complex internalization and degradation) is used in the MM model and incorporates *k*_on_, *k*_off_, and *k*_int_.

This PBPK model with TMDD for efalizumab was verified by comparison of the predicted concentration-time outputs for single and multiple doses with observations from the following clinical trials:

1 mg/kg – Bauer et al. (12);
3 mg/kg – Bauer et al. (12) and Ng et al. (18);
10 mg/kg – Bauer et al. (12);
multiple dosing: 0.3 mg/kg/week for week 1, 0.4 mg/kg/week for week 2;
0.6 mg/kg/week for week 3 and 1 mg/kg/week for the next 4 weeks – Gottlieb;
1 mg/kg/week – Ng et al. (18).

Reported mean data from these studies were obtained by digitization (Digidata^®^).

Relevant predicted and observed pharmacokinetic parameters were also compared.

To demonstrate the relevance of TMDD on the disposition of mAbs, PK of the three single doses were compared with and without TMDD.

Free CD11a levels were used to evaluate the PD effects of efalizumab. Predicted changes in CD11a concentrations as *a*% of baseline concentrations (at time 0 h) were compared with those observed clinically (14).

### PBPK/PD model to simulate mAb efficacy

An integrated response model was constructed to predict the changes in mean PASI scores that were measured in a clinical study (14), as an efficacy marker for efalizumab treatment. This was based on the following mathematical relationship, where *Y* (*t*) is the rate of progression of the disease, *Y*_ss_ is the steady state asymptote, and *T*_p_ is the half-life of progression (24):

$$Y\left(t\right)=Y_{\mathrm{ss}}+\left(Y\left(0\right)-Y_{\mathrm{ss}}\right)e^{-\frac{\ln2}{T_{p}}}\times t$$

Psoriasis Area and Severity Index scores were approximated as continuous and following the first-order asymptotic progression model with baseline score (*Y*_0_) modulated in proportion to concentration of bound CD11a. Both the baseline PASI score (*Y*_0_) and the steady state asymptote (*Y*_ss_) were obtained from a clinical study (14). The half-life of progression of the psoriasis (*T*_p_) in this model was obtained by parameter estimation.

The study design used by Gottlieb and coworkers (14) was used for initial model construction. The model corresponded to their study arm in which patients with moderate to severe psoriasis (mean baseline PASI score of 24.8 – CV% 10.8) were given an escalating dosage regimen of efalizumab (0.3 mg/kg in week 1; 0.4 mg/kg in week 2; 0.6 mg/kg in week 3; and 1 mg/kg/week for the following 4 weeks) by infusion (1 h). PASI scores were assessed weekly and a mean score of approximately 14.8 (CV% 22) was observed in the last three consecutive weeks. This value was used as the *Y*_ss_ in the model. The constructed model was tested for its ability to recover the changes in PASI scores observed clinically by visual comparisons of the mean response–time curves.

The applicability of this model to predict mAb efficacy in other patient cohorts was then evaluated. The mean baseline was changed to 19, corresponding to that in the study by Gordon et al. (16). Since a 1 mg/kg/week given by iv infusion usually produces a change in PASI score from baseline of about 45–50%, *Y*_ss_ was given a value of 9.5. The predicted response over time was then compared with that observed clinically. The simulation was considered to be adequate when the observed data points were within the predicted 5th and 95th centile.

### Simulations

Simulations were conducted with five trials using virtual north European Caucasian Healthy Volunteers. For replication of clinical observations, simulations were based on trial designs that were as close as possible to the clinical study design. Predictive studies used 5 trials with 100 virtual north European Caucasian Healthy Volunteers each, aged between 25 and 50 years, with an equal proportion of males and females. A visual comparison was done between predicted and observed concentration–time profiles and response–time profiles to verify suitability of the models. The simulation was considered to be adequate when the observed data points were within the predicted 5th and 95th centile.

## Results

### PK of single and multiple doses of efalizumab

**Figure 2:**
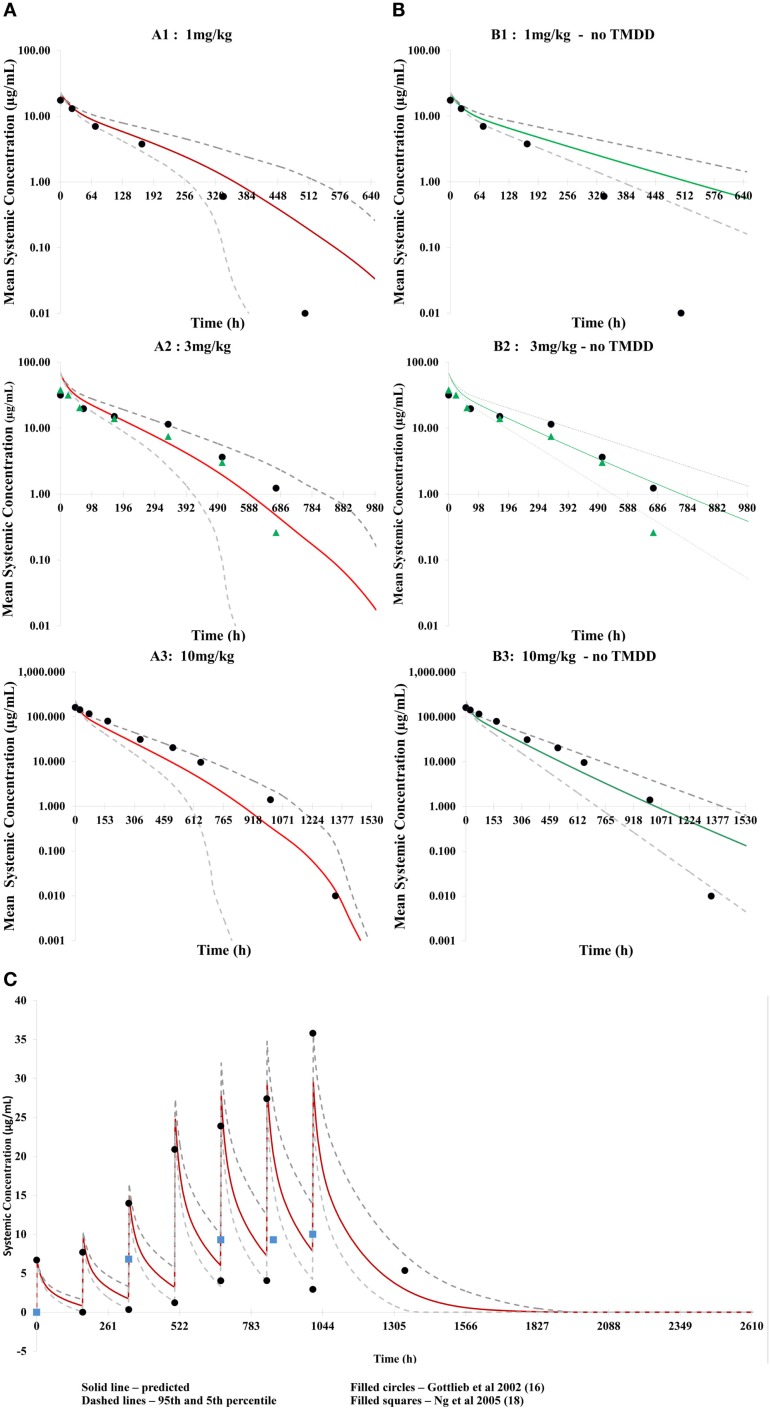
**Predicted concentration–time profiles for three different single doses of efalizumab, comparing the concentration–time profiles with TMDD in the model [graphs (A): (A1–A3)] and without TMDD [graphs (B): (B1–B3)]**. The prediction is improved by TMDD since more of the clinically observed values lie within the 5–95 percentiles of the prediction (broken lines). Predicted concentration–time profile for multiple dosing with TMDD is shown in **(C)**.

Application of the mechanistic FcRn model with TMDD predicted concentration–time profiles for efalizumab 1, 3, and 10 mg/kg adequately, as can be seen in Figure 2A. Corresponding predicted versus observed clearance values are shown in Table 2 and indicate that the model was able to predict PK adequately.

**Table 2:** **Predicted and observed CL**.

Dose (mg/kg)	Observed CL (ml/day/kg); Mean (range)	Predicted CL (ml/day/kg); Mean (range)	Predicted CL (ml/day/kg); Mean (range)
	
		With TMDD	Without TMDD
1	15.6^a^ (10.6–20.6)	11.65 (10.3–11.8)	9.23 (8.2–9.5)
3	10.7^a^ (7.1–13.3)	10.09 (8.9–10.2)	9.01 (7.9–9.1)
10	6.64^a^^,^^b^	9.3 (8.3–9.5)	9.01 (7.9–9.07)

*^a^Ref. (12)*.

*^b^Result from one subject*.

Application of the model for prediction of PK using multiple dosing also recovered clinical data from Gottlieb et al. (15) and Ng et al. (18) adequately (Figure 2C).

### TMDD effects on mAb disposition

Differences in the PK of efalizumab, in the absence of binding to CD11a, are shown in Figure 2B. The lower clearance values obtained in the absence of TMDD are shown in Table 2. Results suggest that TMDD is more important for lower drug concentrations.

### Prediction of free CD11a concentrations as *a*% of the baseline during efalizumab treatment

**Figure 3:**
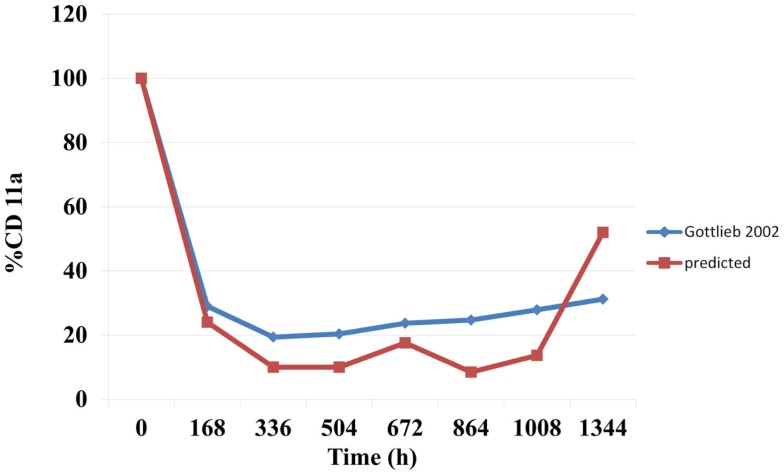
**Predicted and observed changes in CD11a concentration expressed as a % of baseline**.

The predicted suppression of CD11a concentrations that were sustained over the treatment period are shown in Figure 3. Mean predicted CD11a% were marginally lower than those observed in the study by Gottlieb et al. (15).

### PBPK linked PD model

**Figure 4:**
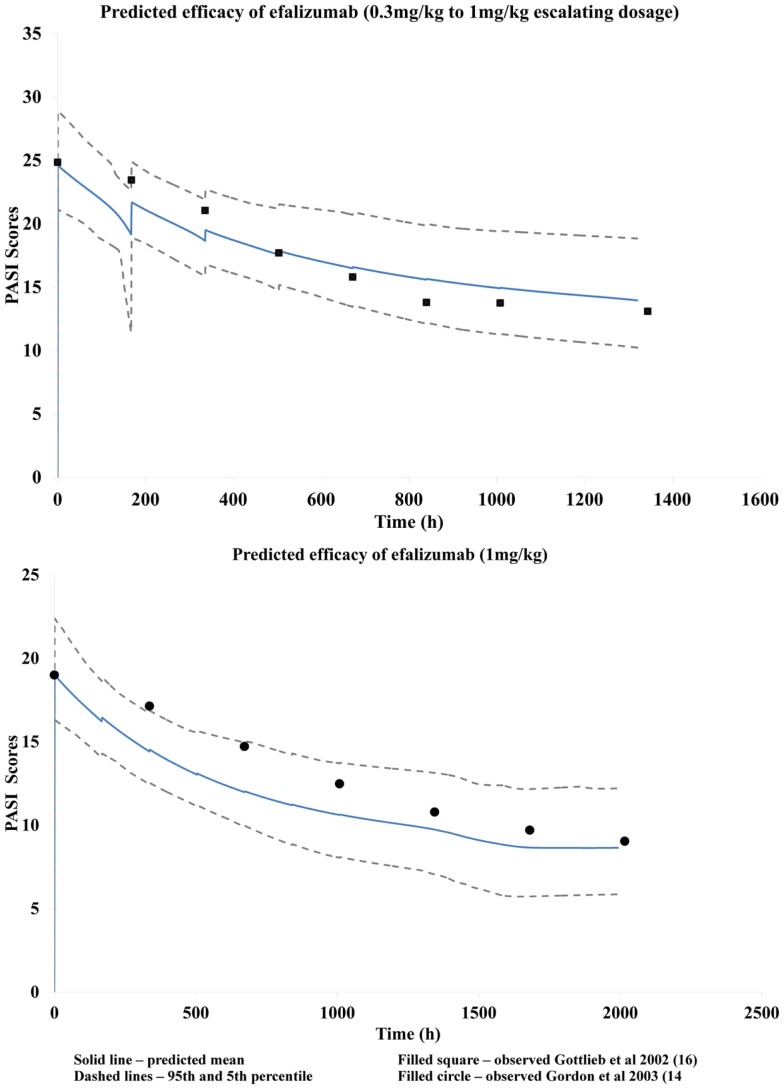
**Predicted changes in PASI scores during the treatment period**.

A fitted *T*_p_ parameter value of 397 h was obtained using parameter estimation module of the Simulator and the clinical data by Gottlieb et al. (15) Visual predictive checks suggested that the resulting PBPK/PD model was reasonably successful at recovering the changes in PASI scores over time as observed by Gottlieb et al. (15) using escalating dosage (Figure 4). This developed model was then used successfully to predict response observed clinically by Gordon et al. (16) for a dose of 1 mg/kg (Figure 4) by using just the mean baseline score of the patients in the latter clinical study.

## Discussion

A PBPK model with a linked PD model that successfully predicted the pharmacokinetics, immunobiologic, and efficacy of a mAb, was developed in this study. Although a limited number of PBPK models that predict mAb PK have been published, PBPK models that predict pharmacokinetics as well as PD and efficacy in humans are uncommon.

The concentration–time profiles of efalizumab in Figure 2 suggest that this model predicts the PK of single as well as multiple doses of efalizumab reasonably well. Visual inspection of the concentration–time profiles for 1, 3, and 10 mg/kg suggested a reasonable recovery of the clinical data, with all data points within the 95th and 5th percentile. A comparison of the predicted and observed clearance values (Table 2) show that the predicted values for 1 and 3 mg/kg are within the observed ranges. Predicted clearance for 10 mg/kg was higher than the clearance reported by Bauer et al. (12), but only one patient received that dose in the Bauer study. The concentration–time profile following multiple dosing was also favorable predicted as seen in Figure 2C. Gottlieb and coworkers (14) measured peak (*C*_max_) and trough (*C*_min_) concentrations following a dosing regimen that escalated from 0.3 mg/kg in week 1 to 1 mg/kg in week 4. The simulation in this study used a similar dosing regimen and was able to recover the observed data adequately. Ng and coworkers (18) reported concentrations following a dose of 1 mg/kg/week. In general, the model was successful at predicting the disposition of efalizumab.

Efalizumab clearance was influenced by TMDD. Binding to CD11a produced the desired therapeutic effects of efalizumab but also lead to internalization of the drug–target complex and catabolism. This was a highly specific clearance process because of the target specificity and was also saturable, based on free target availability. The model accounted for changing concentrations of the target (due to synthesis and degradation). Thus, the mechanistic FcRn model coupled with the Michaelis–Menten approximation of the TMDD model (23) adequately predicted the disposition of efalizumab.

The reduced ability of the model without TMDD to predict concentration–time profiles for efalizumab (especially at lower doses – Figure 2B) illustrates the importance of TMDD in the PK of efalizumab. It is suggested that at higher mAb concentrations, the target concentrations may be saturated, making the TMDD pathway less important in clearance.

The binding of mAbs to specific targets for their therapeutic activity make this therapeutic class attractive to immunologists and clinicians. Efalizumab, which was indicated for the treatment of psoriasis, is known as an anti-CD11a drug since its binds to CD11a, a cell surface receptor. Efalizumab binds to CD11a, which is the α subunit of LFA-1, thereby interrupting the T lymphocyte-mediated actions and alleviating the symptoms of psoriasis. Clinical studies have shown a significant downregulation of CD11a concentrations (typically to about 25% of baseline) (18) during efalizumab administration. A sustained downregulation of CD11a has been accompanied by favorable reductions in PASI scores. The model in this study predicted the suppression of the CD11a concentrations (Figure 3). These were marginally lower than those observed by Gottlieb et al. (15). However, significant inter-individual variability has been reported for CA11a (18).

The disease progression model used to characterize this chronic autoimmune disease accounts for the symptomatic relief by efalizumab as shown by the improvement in PASI scores. Efalizumab modulates the baseline PASI score, i.e., *Y* (0), which corresponds to symptomatic effect, without having any effect on the half-life of the disease progression (*T*_p_). The model recovered clinically observed changes in PASI corresponding to efalizumab treatment reasonably well, as seen in Figure 4. Use of this model to predict changes in PASI scores over time for other patient groups, as in the case of the clinical study by Gordon et al. (16), illustrates the robustness of the model and its potential utility during drug development and clinical practice. This study suggests that a PBPK/PD model that is developed for a mAb may be used to make predictions based on different population groups, disease severity, and perhaps different dosages and formulations. The model also has the potential for application to other mAbs in the same therapeutic class, for comparison of efficacy.

Although reasonably good predictions of PK, PD, and efficacy were obtained using this PBPK linked PD model, some opportunities may exist for improvement of the model. Firstly, parameterization of the PBPK model is generally problematic because of the limited data and the general lack of consensus on PBPK models for mAbs, especially in humans. The variability on the parameters in the disease progression model may also be a limitation, since mean data were obtained from published studies and variability could not be accurately assessed. This model does not account for anti-CD11a antibodies. While the PD model predicted the PASI scores over time for the two studies and different doses, application of this model to other compounds will require some knowledge of the expected maximum change in PASI score for the compound. In addition, this model is not designed to simulate placebo effects of the drug.

Based on the acceptable predictions of efalizumab clearance, concentration–time profiles, CD11a suppression and simulations of PASI score changes over the treatment period, it can be concluded that the study was successful in developing a PD model linked to a mechanistic FcRn PBPK model to predict PK, PD, and efficacy of mAbs in humans. Similar models can be constructed for “testing” various “what/if” scenarios during mAb development and thereby inform the designs of clinical studies. PBPK models provide the opportunity for simulation of various scenarios that may not be included in initial clinical trials such as differences in FcRn abundances, target concentrations, patients of different ethnicities, special population groups, as well as mAbs with variable affinities for FcRn or the target.

## Author Contributions

Manoranjenni Chetty designed and performed the research and analyzed the data. Manoranjenni Chetty, Linzhong Li, Rachel Rose, Krishna Machavaram, Masoud Jamei, Amin Rostami-Hodjegan, and Iain Gardner wrote the manuscript.

## Conflict of Interest Statement

Manoranjenni Chetty, Linzhong Li, Rachel Rose, Krishna Machavaram, Iain Gardner, and Masoud Jamei are employees of Simcyp Limited (a Certara company). Amin Rostami-Hodjegan is an employee of the University of Manchester and part-time secondee to Simcyp Limited (a Certara Company).
